# Air pollution by NO_2_ is associated with the risk of Bell’s palsy: A nested case-controlled study

**DOI:** 10.1038/s41598-020-61232-7

**Published:** 2020-03-06

**Authors:** So Young Kim, Chanyang Min, Jay Choi, Bumjung Park, Hyo Geun Choi

**Affiliations:** 1 Department of Otorhinolaryngology-Head & Neck Surgery, CHA Bundang Medical Center, CHA University, Seongnam, Korea; 2 Hallym Data Science Laboratory, Hallym University College of Medicine, Anyang, Korea; 3 Graduate School of Public Health, Seoul National University, Seoul, Korea; 4 Department of Otorhinolaryngology-Head & Neck Surgery, Hallym University College of Medicine, Anyang, Korea; 5 Hallym Convergence Research Institute for Environmental Diseases, Anyang, Korea

**Keywords:** Peripheral neuropathies, Risk factors

## Abstract

This study investigated the relationship of weather and air pollution with the onset of Bell’s palsy. The Korean Health Insurance Review and Assessment Service-National Sample Cohort (HIRA-NSC) data from 2002 through 2013 were used. The 3,935 Bell’s palsy patients were matched with 15,740 control participants. The meteorological data, including daily mean temperature (°C), daily mean highest temperature (°C), daily mean lowest temperature (°C), daily mean temperature difference (°C), relative humidity (%), spot atmospheric pressure (hPa), sulfur dioxide (SO_2_) (ppm), nitrogen dioxide (NO_2_) (ppm), ozone (O_3_) (ppm), carbon monoxide (CO) (ppm), and PM_10_ (particulate matter ≤ 10 μg/m^3^) for 60 days, 30 days, 14 days, 7 days, and 3 days prior to the index date were analyzed for Bell’s palsy cases and controls. Conditional logistic regression analysis was used to estimate the odds ratios (ORs) of the association between the meteorological data and Bell’s palsy. The mean NO_2_ and PM_10_ concentrations for 60 days were higher, while that of O_3_ was lower in the Bell’s palsy group than in the control group (both P < 0.001). The Bell’s palsy group showed 16.63-fold higher odds of NO_2_ for 60 days (0.1 ppm) than the control group (95% CI = 10.18–27.16, P < 0.001). The ORs of PM_10_, and O_3_ for 60 days showed inconsistent results according to the included variables. Bell’s palsy was related to high concentrations of NO_2_.

## Introduction

Bell’s palsy is defined as idiopathic peripheral facial paralysis^1^. The incidence of Bell’s palsy is approximately 11–40 per 100,000 person/year worldwide^1^. In Korea, approximately 0.12% of the population, regardless of age, suffers from facial palsy^2^. Viral infection and vascular compromise are thought to be related to Bell’s palsy^3^. Because both viral and cardiovascular causes might be influenced by meteorological factors, such as temperature and air pollution^4–7^, meteorological factors may have indirect effects on Bell’s palsy. In Korea, there is a traditional assumption that cold exposure causes Bell’s palsy^8,9^.

However, previous studies reported conflicting results regarding the association of Bell’s palsy and cold exposure^3,10–13^. Some researchers reported a high incidence of Bell’s palsy in cold weather^10,11^. On the other hand, others demonstrated a high incidence of Bell’s palsy in warm weather or no difference in incidence based on the weather conditions^3,12,13^. Most previous studies based their analysis on regional incidence rates without the consideration of individual factors, and they had small study populations^3,10–13^. To the best of our knowledge, no previous study has evaluated the effect of air pollution on Bell’s palsy. When the PubMed and EMBASE databases were searched through November 2019 for studies using the keyword phrase ‘(Bell’s palsy) AND (air pollution)’, two papers were identified; however, neither was relevant^13,14^.

The hypothesis of the present study was that meteorological conditions, including air pollution, could increase the incidence of Bell’s palsy. To test this hypothesis, the exposures of patients with Bell’s palsy and a control group to meteorological conditions were compared.

## Results

The demographic factors, hypertension, diabetes, and dyslipidemia were identical between the Bell’s palsy and control groups. The mean meteorological and air pollution measurements for 60 days before the index date were evaluated. Only nitrogen dioxide (NO_2_), ozone (O_3_), and PM_10_ (particulate matter ≤10 μg/m^3^) showed differences (Table 1, each of P < 0.001).

**Table 1:** General characteristics of participants.

Characteristics	Total participants		
	Bell’s palsy	Control group	P-value
Age (years old, n, %)			1.000
0–4	19 (0.5)	76 (0.5)	
5–9	25 (0.6)	100 (0.6)	
10–14	81 (2.1)	324 (2.1)	
15–19	95 (2.4)	380 (2.4)	
20–24	127 (3.2)	508 (3.2)	
25–29	215 (5.5)	860 (5.5)	
30–34	254 (6.5)	1,016 (6.5)	
35–39	307 (7.8)	1,228 (7.8)	
40–44	316 (8.0)	1,264 (8.0)	
45–49	409 (10.4)	1,636 (10.4)	
50–54	463 (11.8)	1,852 (11.8)	
55–59	450 (11.4)	1,800 (11.4)	
60–64	362 (9.2)	1,448 (9.2)	
65–69	327 (8.3)	1,308 (8.3)	
70–74	229 (5.8)	916 (5.8)	
75–79	151 (3.8)	604 (3.8)	
80–84	79 (2.0)	316 (2.0)	
85+	26 (0.7)	104 (0.7)	
Sex (n, %)			1.000
Male	1,848 (47.0)	7,392 (47.0)	
Female	2,087 (53.0)	8,348 (53.0)	
Income (n, %)			1.000
1 (lowest)	79 (2.0)	316 (2.0)	
2	268 (6.8)	1,072 (6.8)	
3	256 (6.5)	1,024 (6.5)	
4	244 (6.2)	976 (6.2)	
5	287 (7.3)	1,148 (7.3)	
6	310 (7.9)	1,240 (7.9)	
7	354 (9.0)	1,416 (9.0)	
8	394 (10.0)	1,576 (10.0)	
9	503 (12.8)	2,012 (12.8)	
10	559 (14.2)	2,236 (14.2)	
11 (highest)	681 (17.3)	2,724 (17.3)	
Region of residence (n, %)			1.000
Urban	1,779 (45.2)	7,116 (45.2)	
Rural	2,156 (54.8)	8,624 (54.8)	
Hypertension (n, %)	1,602 (40.7)	6,408 (40.7)	1.000
Diabetes (n, %)	977 (24.8)	3,908 (24.8)	1.000
Dyslipidemia (n, %)	1,229 (31.2)	4,916 (31.2)	1.000
Daily mean temperature for 60 days (°C, mean, SD)	12.6 (9.3)	12.8 (9.2)	0.444
Daily highest temperature for 60 days (°C, mean, SD)	17.8 (9.1)	18.0 (9.0)	0.439
Daily lowest temperature for 60 days (°C, mean, SD)	8.2 (9.7)	8.3 (9.6)	0.476
Daily temperature difference for 60 days (°C, mean, SD)	9.6 (2.0)	9.6 (1.9)	0.956
Relative humidity for 60 days (%, mean, SD)	65.7 (9.2)	65.7 (9.5)	0.973
Spot atmospheric pressure for 60 days (hPa, mean, SD)	1006.5 (7.1)	1006.3 (7.4)	0.191
SO_2_ for 60 days (ppb, mean, SD)	5.5 (1.8)	5.6 (1.8)	0.087
NO_2_ for 60 days (ppb, mean, SD)	25.4 (8.3)	23.9 (7.9)	<0.001*
O_3_ for 60 days (ppb, mean, SD)	22.4 (7.8)	23.1 (7.8)	<0.001*
CO for 60 days (ppm, mean, SD)	0.578 (0.173)	0.572 (0.174)	0.087
PM_10_ for 60 days (μg/m^3,^ mean, SD)	52.9 (13.9)	52.0 (13.4)	<0.001*

SD: standard deviation.

ppb: Parts per billion.

ppm: Part per million (=1,000 ppb).

*Chi-square test or independent T-test. Significance at P < 0.05.

The odds ratio (OR) of the 60-day NO_2_ exposure level (0.1 ppm) prior to the onset of Bell’s palsy was 16.63 (95% CI = 10.18–27.16, P < 0.001, Table 2). The OR of the 60-day PM_10_ exposure level (10 μg/m^3^) prior to the onset of Bell’s palsy was 1.07 (95% confidence interval [95% CI] = 1.04–1.11, P < 0.001), while that of O_3_ was 0.18 (95% CI = 0.10–0.31, P < 0.001). The daily mean temperature, daily mean highest temperature, daily mean lowest temperature, daily mean temperature difference, relative humidity, spot atmospheric pressure, SO_2_, and CO were not different between the Bell’s palsy and control groups (S1 Table). After performing several analyses of the associations between various levels of exposure to NO_2_, O_3_, and PM_10_ and Bell’s palsy, 60-day exposure levels of NO_2_, O_3_, and PM_10_ prior to the onset of Bell’s palsy were selected based on the Akaike information criterion (AIC) and Bayesian information criterion (BIC) values (S2 Table).

**Table 2:** Adjusted odd ratios, 95% confidence interval, Akaike information criterion and Baysian information criterion of the pollution matters in conditional logistic regression for FNP.

Pollution matters	OR (95% CI)	P-value	AIC	BIC
Model 1			15458.61	15466.49
NO_2_ for 60 days (0.1 ppm)	16.63 (10.18–27.16)	<0.001*		
Model 2			15547.66	15555.55
O_3_ for 60 days (0.1 ppm)	0.18 (0.10–0.31)	<0.001*		
Model 3			15562.98	15570.87
PM_10_ for 60 days (10 μg/m^3^)	1.07 (1.04–1.11)	<0.001*		
Model 4			15460.59	15476.37
NO_2_ for 60 days (0.1 ppm)	16.35 (9.14–29.26)	<0.001*		
O_3_ for 60 days (0.1 ppm)	0.97 (0.50–1.87)	0.915		
Model 5			15453.41	15469.18
NO_2_ for 60 days (0.1 ppm)	27.77 (14.97–51.52)	<0.001*		
PM_10_ for 60 days (10 μg/m^3^)	0.95 (0.91–0.99)	0.007*		
Model 6			15535.76	15551.53
O_3_ for 60 days (0.1 ppm)	0.21 (0.12–0.37)	<0.001*		
PM_10_ for 60 days (10 μg/m^3^)	1.06 (1.03–1.10)	<0.001*		
Model 7			15455.10	15478.77
NO_2_ for 60 days (0.1 ppm)	31.21 (14.82–65.72)	<0.001*		
O_3_ for 60 days (0.1 ppm)	1.21 (0.61–2.40)	0.582		
PM_10_ for 60 days (10 μg/m^3^)	0.94 (0.91–0.98)	0.006*		

CI: confidence interval.

AIC: Akaike information criterion.

BIC: Baysian information criterion.

*Conditional logistic regression was performed. Models were stratified by age, sex, income, region of residence, hypertension, diabetes, and dyslipidemia. Significance at P < 0.05.

Model 4: adjusted for NO_2_ and O_3_.

Model 5: adjusted for NO_2_ and PM_10_.

Model 6: adjusted for O_3_ and PM_10_.

Model 7: adjusted for NO_2,_ O_3_, and PM_10_.

In the various models (models 1–7, Table 2), the results for NO_2_ were consistent, while those of O_3_ and PM_10_ were changed according to the variables included in the different models.

In the subgroup analyses, the 60-day exposure level of NO_2_ (0.1 ppm) was associated with an increased risk of Bell’s palsy in young women (OR = 68.17, 95% CI = 10.94–424.57, P < 0.001), middle-aged men (OR = 27.84, 95% CI = 11.22–69.10, P < 0.001), middle-aged women (AOR = 12.53, 95% CI = 4.96–31.66, P < 0.001), old men (OR = 12.93, 95% CI = 2.87–58.24), and old women (OR = 20.05, 95% CI = 6.20–64.82) groups (Table 3). However, statistical significance was not reached in the group of young men. According to the region of residence, there was an association between the exposure to NO_2_ with an elevated risk of Bell’s palsy in both urban and rural residents (S3 Table).

**Table 3:** Adjusted odd ratios (95% confidence interval) of NO_2_ for 60 days (0.1 ppm) for Bell’s palsy in subgroup analysis according to age and sex.

Subgroup	N (participants)	Bell’s palsy	
		OR of NO_2_	P-value
Total	19,675	16.63 (10.18–27.16)	<0.001*
Age (<30 years old), men	1,425	1.56 (0.26–9.36)	0.629
Age (<30 years old), women	1,385	68.17 (10.94–424.57)	<0.001*
Age (30–59 years old), men	5,595	27.84 (11.22–69.10)	<0.001*
Age (30–59 years old), women	5,400	12.53 (4.96–31.66)	<0.001*
Age (≥60 years old), men	2,220	12.93 (2.87–58.24)	0.001*
Age (≥60 years old), women	3,650	20.05 (6.20–64.82)	<0.001*

*Conditional logistic regression was performed. Models were stratified by age, sex, income, region of residence, hypertension, diabetes, and dyslipidemia. Significance at P < 0.05.

## Discussion

The concentrations of NO_2_ for 60 days before the onset of Bell’s palsy were higher in Bell’s palsy patients than in the control group in this study. Other meteorological factors, such as temperature, humidity, atmospheric pressure, SO_2_, and CO, were not associated with Bell’s palsy. The results for O_3_ and PM_10_ were inconsistent (S4 Table). To the best of our knowledge, there has been no study on the association of air pollutants with Bell’s palsy.

The oxidative stress and inflammatory response to NO_2_ exposure may directly influence the development of Bell’s palsy. The cumulative effects of high concentrations of NO_2_ might contribute to the increased risk of Bell’s palsy. It was reported that patients with Bell’s palsy had higher levels of oxidative stress and antioxidant activity than patients in the control group^15–17^. Patients with Bell’s palsy had increased blood levels of thiol and disulfide activity levels compared to control participants^16^. In addition, the serum levels of malondialdehyde and the antioxidants glutathione, catalase, and superoxide dismutase were elevated in Bell’s palsy patients^17^. The increased level of oxidative stress may elevate an individual’s susceptibility to inflammatory neuropathy. Bell’s palsy is likely accompanied by an inflammatory response. The neutrophil-to-lymphocyte ratio was found to be higher in Bell’s palsy patients, and the ratio was correlated with the House-Brackmann grade of facial palsy and facial nerve enhancement on temporal gadolinium-enhanced magnetic resonance imaging^18^. NO_2_ can oxidize other organic compounds, including unsaturated fatty acids, thereby inducing free radical reactions^19^. The expression of numerous genes related to oxidative stress, including heme-oxygenase 1, was increased after NO_2_ exposure in primary human bronchial epithelial cells^20^. Moreover, systemic inflammation can be induced by NO_2_ exposure. A previous study reported that the serum interleukin-6 concentration was increased 1.20-fold after relatively higher levels of NO_2_ exposure (95% CI = 1.1–1.3, P = 0.001)^21^.

The elevated cardiovascular risk due to NO_2_ might represent an indirect link between NO_2_ and the risk of Bell’s palsy. NO_2_ is known to be associated with the risk of cardiovascular diseases, such as stroke and myocardial infarction, and cardiovascular mortality^22,23^. A meta-analysis study reported that there was increased cardiovascular mortality following long-term NO_2_ exposure (hazard ratio = 1.03, 95% CI = 1.02–1.05)^23^. Among several air pollutants, namely, CO, NO_2_, PM_10_, PM _2.5_, and SO_2_, only NO_2_ was associated with an increased risk of hospital admissions for non-myocardial infarction-related cardiovascular disease (2.0%, 95% CI = 1.1–2.9) and heart failure (4.4%, 95% CI = 2.0–6.8)^24^. Another longitudinal follow-up study demonstrated an excess relative risk of hospital admissions of 2.8% for myocardial infarction and 4.9% for hemorrhagic stroke^22^. Several epidemiological studies have suggested the association of cardiovascular diseases with Bell’s palsy^25,26^. The incidence of peripheral arterial occlusive disease was 1.5 times higher in Bell’s palsy patients than in the control group^25^. Patients with Bell’s palsy had a 2.02-fold higher risk of stroke than the control group (95% CI = 1.42–2.86)^26^.

Similarly, PM_10_ was found to induce oxidative damage by increasing the intracellular level of hydrogen peroxide and decreasing catalase activity in human lung epithelial A549 cells^27^. In addition, PM suppressed the anti-inflammatory functions and innate immune neutrophils to endotoxins of lipopolysaccharides^28^. PM_10_ also had detrimental effects on cardiovascular diseases. A 10 µg/m^3^ increase in PM_10_ concentration was related to a 10.10% increase in the incidence of ST-elevation myocardial infarction^29^. This increase in the incidence of cardiovascular disease could be related to the risk of Bell’s palsy. However, we did not find a consistent result of association between PM_10_ and Bell’s palsy after adjustment for NO_2_ and O_3_. There was no previous study evaluated the relation between PM_10_ and Bell’s palsy, as far as our knowledge. However, possible explanations of the inconsistency in this study include the higher impact of NO_2_ than that of PM_10_ on the risk of Bell’s palsy and relatively small differences on the PM_10,_ which could attenuate the statistical power. More studies are required to explore the relationship between PM_10_ and Bell’s palsy.

Because we could not find any previous study that evaluated the association between O_3_ and Bell’s palsy, we were unable to explain their inverse association. We believe this might be affected by the negative relationship between NO_2_ and O_3,_ as O_3_ could be created from NO_2_ by photolysis via ultraviolet light^30^. Actually, the relative concentration of NO_2_ is higher than that of O_3_ in Korea^31^. Therefore, the dominant effect of NO_2_ on Bell’s palsy might be able to conceal the effects of O_3_ on Bell’s palsy. On the other hand, there is a possibility that O_3_ might actually reduce the risk of Bell’s palsy, as ozone therapy has been reported to affect facial nerve palsy^32^.

Other meteorological conditions, including temperature, atmospheric pressure, and humidity, did not show associations with Bell’s palsy in the present study. In accordance with our results, some prior studies demonstrated that there was no association between weather conditions and the onset of Bell’s palsy^13,33^. On the other hand, other previous studies reported relationships between Bell’s palsy and low temperature and atmospheric pressure^34–36^. However, many previous studies did not assess individual-level factors, such as comorbidities. Instead, these studies only analyzed the regional incidence of Bell’s palsy according to seasons or months^34–36^. In addition, the effects of air pollutants could be influenced by weather conditions. For instance, the synergistic effects of temperature and air pollutants on mortality due to cardiovascular or respiratory diseases have been reported^37,38^. Thus, air pollutants should be included when evaluating the effects of weather conditions on health outcomes. However, most previous studies did not concurrently consider air pollutants and weather conditions.

Unlike previous studies, this study matched and adjusted for individual-level demographic factors and past medical histories. The region of residence was matched between the Bell’s palsy group and the control group. In addition, the subgroup analyses according to region of residence demonstrated consistently high ORs for the association of the risk of Bell’s palsy with NO_2_ exposure in both the urban and rural groups (Table S3). A number of weather conditions, such as temperature, humidity, and atmospheric pressure, and the air pollutants, namely, SO_2_, NO_2_, O_3_, CO, and PM_10_, were simultaneously analyzed. There are several additional merits of the present study. With regard to the meteorological conditions, the accuracy of the measurements was guaranteed by using an automated synoptic observing system and a manual system hourly in 94 and 273 places, respectively (Supplementary S5). In addition, longitudinal measurements extending 60 days before the index dates were analyzed. The best fit models for the associations of NO_2_, O_3_, and PM_10_ air pollutants with Bell’s palsy were identified with the AIC and BIC (Table S2). Moreover, the large, representative sample cohort enabled the analysis of a number of meteorological conditions with satisfactory statistical power. A large, nationwide, representative study was carried out based on National Health Insurance System (NHIS) data. Because all Korean citizens are legally registered and their medical records are managed by the NHIS, no participants were missing in the present study.

However, because the NHIS data are based on hospital or clinical visits, it is possible that subclinical cases could have been excluded from the present study. In addition, although there were numerous participants, the number of young (<30 years old) patients was relatively smaller than the number of older patients, which could be a cause of the nonsignificant association between exposure to air pollutants and Bell’s palsy in this age group (Table 3). Although we considered several demographic factors and comorbidities, there are still possible confounding factors that were not considered, including smoking and alcohol consumption. PM_2.5_ information was not available in this study because it was only measured after 2015 in Korea. Moreover, the intrinsic limitation of the analysis of meteorological exposure in an epidemiological study existed because the measures of meteorological exposures were based on residential areas and could not account for indoor exposure. However, outdoor exposure to nitrogen is thought to have a higher impact on health outcomes than indoor exposure^39^. Lastly, because the Bell’s palsy group was matched with a control group who lived in another urban/rural place, the other environmental factors specific to the respective residential areas, including air pollution or meteorological factors, could have influenced the increased risk of Bell’s palsy.

In conclusion, the exposure to NO_2_ for 60 days before the onset of Bell’s palsy was higher in Bell’s palsy patients than in the control group, while the results for exposure to O_3_ and PM_10_ were inconsistent. Other meteorological conditions, including temperature, humidity, atmospheric pressure, SO_2_, and CO, were not related to the onset of Bell’s palsy.

## Materials and Methods

### Participant Selection

This study was approved by the ethics committee of Hallym University (2017-I102). All methods were performed according the guidelines and regulations of the ethics committee of Hallym University. Written informed consent was waived by the Institutional Review Board. We describe the Korean Health Insurance Review and Assessment Service - National Sample Cohort (HIRA-NSC), meteorological data, and air pollution data in supplemental file 5.

**Figure 1:**
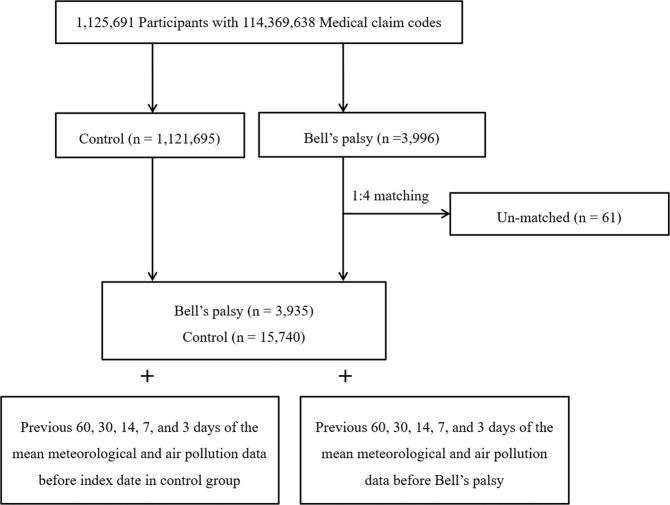
Schematic illustration of the participant selection process that was used in the present study. Out of a total of 1,125,691 participants, 3,935 of Bell’s palsy participants were matched with 15,740 control participants for age, sex, income, region of residence, and past medical histories. Then, the Bell’s palsy and control participants were matched with the same meteorological data before the index date.

From a total of 1,125,691 patients with 114,369,638 medical claim codes, the participants who were defined as having Bell’s palsy were selected for inclusion (n = 3,996). The Bell’s palsy participants were matched 1:4 with participants who had never been diagnosed with Bell’s palsy (control group). Age group, sex, income group, region of residence, and past medical histories (hypertension, diabetes, and dyslipidemia) were matched between the Bell’s palsy and control groups. In matching the region of residence (urban/rural), participants who lived in urban areas were matched with control participants who lived in another urban place. The same method was used for those who lived in rural areas. The control group participants were sorted using a random number generator. The matched control participants were presumed to be enrolled concurrently with each Bell’s palsy participant (index date). The Bell’s palsy participants for whom we could not identify sufficient numbers of matching participants were excluded (n = 61). Finally, 1:4 matching resulted in the inclusion of 3,935 Bell’s palsy participants and 15,740 control participants (Fig. 1).

We analyzed the meteorological data for the mean exposures 60 days, 30 days, 14 days, 7 days, and 3 days before the index date in both Bell’s palsy and control groups.

### Variables

#### Independent variable

The daily mean temperature (°C), daily mean highest temperature (°C), daily mean lowest temperature (°C), daily mean temperature difference (°C), relative humidity (%), spot atmospheric pressure (hPa), sulfur dioxide (SO_2_) (ppm), NO_2_ (ppm), O_3_ (ppm), CO (ppm), and PM_10_ (μg/m^3^) for 14 days, 10 days, 7 days, 5 days, and 3 days before the index date were analyzed (Table S2).

#### Covariates

The age groups were classified using 5-year intervals. The income groups were divided into 11 classes (class 1 [lowest income]-11 [highest income]). The region of residence was classified as urban and rural areas.

The past medical histories of the participants were defined using the 10th revision of the International Statistical Classification of Diseases (ICD-10) codes. Hypertension (I10 and I15), diabetes (E10-E49), and dyslipidemia (E78) were acknowledged if the participants were treated ≥2 times.

#### Dependent variable

Bell’s palsy was defined using the ICD-10 code (G510). Among the cases with this ICD-10 code, the participants who were treated ≥2 times and who were treated with steroids were defined as having Bell’s palsy.

### Statistical Analyses

The rate of general characteristics between Bell’s palsy and the control group were compared using the chi-square test. The mean meteorological data 14 days before the index date was compared using an independent t-test.

To analyze the ORs of meteorological data for Bell’s palsy, conditional logistic regression was performed. The crude (simple) and adjusted (multiple) models were analyzed. The models were stratified by age, sex, income, region of residence, hypertension, diabetes, and dyslipidemia. The 95% CI was described. We describe the independent variables and the methods used to reach the final model in Supplemental Tables 2, 3, and 4.

We estimated a single pollutant model for NO_2,_ O_3_, and PM_10_. Additionally, we calculated a combined model.

The participants were sub-grouped according to age and sex (young [0–29 years old], middle aged [30–59 years old], and elderly [60+ years old]; men, and women). An identical model was used in these analyses.

Two-tailed analyses were performed. and statistical significance was defined as P values less than 0.05. SPSS version 22.0 (IBM, Armonk, NY, USA) and SAS version 9.4 (SAS Institute Inc., Cary, NC, USA) were used to conduct statistical analyses.

## Supplementary information

supplementary tables.
