# Supplementary material for: Air pollution by NO2 is associated with the risk of Bell’s palsy: A nested case-controlled study
Source: Sci Rep. 2020 Mar 6;10:4221. doi: 10.1038/s41598-020-61232-7 (PMC7060183; doi:10.1038/s41598-020-61232-7)
Supplement: Supplementary file 1 — supplementary tables. [file 41598_2020_61232_MOESM1_ESM.docx]

**Air pollution by NO_2_ is associated with the risk of Bell’s palsy:**

**A nested case-controlled study**

**Running title:** Nitrogen dioxide and Bell’s palsy

So Young Kim, MD ^1^, Chanyang Min, PhD ^2,3^, , Jay Choi^1^, Bumjung Park, MD, PhD ^4^, Hyo Geun Choi, MD, PhD ^2,4*^

^1^Department of Otorhinolaryngology-Head & Neck Surgery, CHA Bundang Medical Center, CHA University, Seongnam, Korea

^2^Hallym Data Science Laboratory, Hallym University College of Medicine, Anyang, Korea

^3^Graduate School of Public Health, Seoul National University, Seoul, Korea

^4^Department of Otorhinolaryngology-Head & Neck Surgery, Hallym University College of Medicine, Anyang, Korea

***Correspondence:** pupen@naver.com

**Key words:** Air Pollution; Particulate Matter; Nitrogen Dioxide; Facial Paralysis

**S1 Table** Crude odd ratios (95% confidence interval) of the meteorological and pollution matter for Bell’s palsy

| Characteristics | | Bell’s palsy | |
| --- | --- | --- | --- |
|  |  | OR (95% CI) | P-value |
| Daily mean temperature for 60 days (˚C) | | 1.00 (0.99-1.00) | 0.310 |
| Daily mean temperature for 30 days (˚C) | | 1.00 (0.99-1.00) | 0.347 |
| Daily mean temperature for 14 days (˚C) | | 1.00 (0.99-1.00) | 0.361 |
| Daily mean temperature for 7 days (˚C) | | 1.00 (0.99-1.00) | 0.394 |
| Daily mean temperature for 3 days (˚C) | | 1.00 (0.99-1.00) | 0.378 |
| Daily highest temperature for 60 days (˚C) | | 1.00 (0.99-1.00) | 0.304 |
| Daily highest temperature for 30 days (˚C) | | 1.00 (0.99-1.00) | 0.338 |
| Daily highest temperature for 14 days (˚C) | | 1.00 (0.99-1.00) | 0.352 |
| Daily highest temperature for 7 days (˚C) | | 1.00 (0.99-1.00) | 0.385 |
| Daily highest temperature for 3 days (˚C) | | 1.00 (0.99-1.00) | 0.300 |
| Daily lowest temperature for 60 days (˚C) | | 1.00 (0.99-1.00) | 0.344 |
| Daily lowest temperature for 30 days (˚C) | | 1.00 (0.99-1.00) | 0.386 |
| Daily lowest temperature for 14 days (˚C) | | 1.00 (0.99-1.00) | 0.404 |
| Daily lowest temperature for 7 days (˚C) | | 1.00 (0.99-1.00) | 0.439 |
| Daily lowest temperature for 3 days (˚C) | | 1.00 (0.99-1.00) | 0.474 |
| Daily temperature difference for 60 days (˚C) | | 1.00 (0.98-1.02) | 0.940 |
| Daily temperature difference for 30 days (˚C) | | 1.00 (0.98-1.02) | 0.878 |
| Daily temperature difference for 14 days (˚C) | | 1.00 (0.98-1.02) | 0.869 |
| Daily temperature difference for 7 days (˚C) | | 1.00 (0.98-1.02) | 0.845 |
| Daily temperature difference for 3 days (˚C) | | 0.99 (0.98-1.01) | 0.355 |
| Relative humidity for 60 days (%) | | 1.00 (1.00-1.01) | 0.965 |
| Relative humidity for 30 days (%) | | 1.00 (1.00-1.00) | 0.819 |
| Relative humidity for 14 days (%) | | 1.00 (1.00-1.00) | 0.955 |
| Relative humidity for 7 days (%) | | 1.00 (1.00-1.00) | 0.978 |
| Relative humidity for 3 days (%) | | 1.00 (1.00-1.00) | 0.717 |
| Spot atmospheric pressure for 60 days (hPa) | | 1.01 (1.00-1.01) | 0.123 |
| Spot atmospheric pressure for 30 days (hPa) | | 1.00 (1.00-1.01) | 0.121 |
| Spot atmospheric pressure for 14 days (hPa) | | 1.00 (1.00-1.01) | 0.119 |
| Spot atmospheric pressure for 7 days (hPa) | | 1.00 (1.00-1.01) | 0.145 |
| Spot atmospheric pressure for 3 days (hPa) | | 1.00 (1.00-1.01) | 0.183 |
| SO_2_ for 60 days (0.1 ppm) | | 0.13 (0.01-1.10) | 0.061 |
| SO_2_ for 30 days (0.1 ppm) | | 0.16 (0.02-1.29) | 0.085 |
| SO_2_ for 14 days (0.1 ppm) | | 0.19 (0.03-1.38) | 0.100 |
| SO_2_ for 7 days (0.1 ppm) | | 0.30 (0.04-2.06) | 0.221 |
| SO_2_ for 3 days (0.1 ppm) | | 0.28 (0.05-1.66) | 0.160 |
| NO_2_ for 60 days (0.1 ppm) | | 16.63 (10.18-27.16) | <0.001* |
| NO_2_ for 30 days (0.1 ppm) | | 13.82 (8.58-22.24) | <0.001* |
| NO_2_ for 14 days (0.1 ppm) | | 11.87 (7.52-18.72) | <0.001* |
| NO_2_ for 7 days (0.1 ppm) | | 10.37 (6.74-15.97) | <0.001* |
| NO_2_ for 3 days (0.1 ppm) | | 6.69 (4.51-9.94) | <0.001* |
| O_3_ for 60 days (0.1 ppm) | | 0.18 (0.10-0.31) | <0.001* |
| O_3_ for 30 days (0.1 ppm) | | 0.22 (0.13-0.38) | <0.001* |
| O_3_ for 14 days (0.1 ppm) | | 0.25 (0.15-0.42) | <0.001* |
| O_3_ for 7 days (0.1 ppm) | | 0.28 (0.18-0.46) | <0.001* |
| O_3_ for 3 days (0.1 ppm) | | 0.34 (0.21-0.52) | <0.001* |
| CO for 60 days (1 ppm) | | 1.28 (1.01-1.61) | 0.043* |
| CO for 30 days (1 ppm) | | 1.22 (0.97-1.53) | 0.085 |
| CO for 14 days (1 ppm) | | 1.18 (0.95-1.46) | 0.137 |
| CO for 7 days (1 ppm) | | 1.20 (0.98-1.48) | 0.078 |
| CO for 3 days (1 ppm) | | 1.17 (0.97-1.41) | 0.108 |
| PM_10_ for 60 days (10 μg/m^3^) | | 1.08 (1.04-1.11) | <0.001* |
| PM_10_ for 30 days (10 μg/m^3^) | | 1.06 (1.03-1.09) | <0.001* |
| PM_10_ for 14 days (10 μg/m^3^) | | 1.04 (1.02-1.07) | 0.001* |
| PM_10_ for 7 days (10 μg/m^3^) | | 1.03 (1.01-1.06) | 0.002* |
| PM_10_ for 3 days (10 μg/m^3^) | | 1.02 (1.00-1.04) | 0.020* |

* Conditional logistic regression was performed. Models were stratified by age, sex, income, region of residence, hypertension, diabetes, and dyslipidemia. Significance at P < 0.05

We analyzed the odd ratios of meteorological data for Bell’s palsy using simple logistic regression analysis. In these results, only NO_2,_ O_3_, and PM_10_ showed statistical significance (P < 0.05). Therefore, we choose these NO_2,_ O_3_, and PM_10_ as the independent variables.

**S2 Table** Akaike information criterion and Baysian information criterion of the pollution matters in crude logistic regression for Bell’s palsy

| Pollution matters | AIC | BIC |
| --- | --- | --- |
| NO_2_ for 60 days (0.1 ppm) | 15458.61 | 15466.49 |
| NO_2_ for 30 days (0.1 ppm) | 15468.01 | 15475.90 |
| NO_2_ for 14 days (0.1 ppm) | 15471.97 | 15479.86 |
| NO_2_ for 7 days (0.1 ppm) | 15472.37 | 15480.26 |
| NO_2_ for 3 days (0.1 ppm) | 15496.62 | 15504.50 |
| O_3_ for 60 days (0.1 ppm) | 15547.66 | 15555.55 |
| O_3_ for 30 days (0.1 ppm) | 15553.35 | 15561.24 |
| O_3_ for 14 days (0.1 ppm) | 15556.37 | 15564.25 |
| O_3_ for 7 days (0.1 ppm) | 15558.26 | 15566.14 |
| O_3_ for 3 days (0.1 ppm) | 15561.09 | 15568.98 |
| PM_10_ for 60 days (μg/m^3^) | 15562.98 | 15570.87 |
| PM_10_ for 30 days (μg/m^3^) | 15569.74 | 15577.63 |
| PM_10_ for 14 days (μg/m^3^) | 15573.08 | 15580.97 |
| PM_10_ for 7 days (μg/m^3^) | 15574.49 | 15582.38 |
| PM_10_ for 3 days (μg/m^3^) | 15578.97 | 15586.86 |

AIC: Akaike information criterion

BIC: Baysian information criterion

Because all of NO_2_, O_3_, and PM_10_ (for 60 days, 30 days, 14 days, 7 days, and 3 days) showed statistical significance (S2 table), we had to choose only one of them.

In NO_2_, AIC and BIC showed the smallest value for 60 days. We choose for 60 days before the index date.

In O_3_, for 60 days showed the smallest AIC, and BIC. We chose the means of O_3_ for 60 days before the index date.

In PM_10_, for 60 days showed the smallest AIC, and BIC. We chose the means of PM_10_ for 60 days before the index date.

**S3 Table** Adjusted odd ratios (95% confidence interval) of NO_2_ for 60 days (0.1 ppm) and PM_10_ for 60 days (10 μg/m^3^) for Bell’s palsy in subgroup analysis according to region of residence

| Subgroup | | N (participants) | Bell’s palsy | |
| --- | --- | --- | --- | --- |
|  | |  | AOR | P-value |
| NO_2_ for 60 days | |  |  |  |
|  | Urban | 8,895 | 23.71 (11.50-48.91) | <0.001* |
|  | Rural | 10,780 | 12.30 (6.31-23.98) | <0.001* |
| PM_10_ for 60 days | |  |  |  |
|  | Urban | 8,895 | 1.04 (0.99-1.09) | 0.126 |
|  | Rural | 10,780 | 1.10 (1.06-1.15) | <0.001* |

* Conditional logistic regression was performed. Models were stratified by age, sex, income, region of residence, hypertension, diabetes, and dyslipidemia. Significance at P < 0.05

**S4 Table** Adjusted odd ratios (95% confidence interval) of PM_10_ for 60 days (10 μg/m^3^) for Bell’s palsy in subgroup analysis according to age and sex

| Subgroup | N (participants) | Bell’s palsy | |
| --- | --- | --- | --- |
|  |  | OR of PM_10_ | P-value |
| Total | 19,675 | 1.07 (1.04-1.11) | <0.001* |
| Age (<30 years old), men | 1,425 | 1.03 (0.92-1.15) | 0.651 |
| Age (<30 years old), women | 1,385 | 1.14 (1.01-1.28) | 0.030* |
| Age (30-59 years old), men | 5,595 | 1.09 (1.03-1.15) | 0.005* |
| Age (30-59 years old), women | 5,400 | 1.07 (1.01-1.14) | 0.018* |
| Age (≥ 60 years old), men | 2,220 | 1.11 (0.99-1.24) | 0.068 |
| Age (≥ 60 years old), women | 3,650 | 1.05 (0.98-1.13) | 0.188 |

* Conditional logistic regression was performed. Models were stratified by age, sex, income, region of residence, hypertension, diabetes, and dyslipidemia. Significance at P < 0.05

## **Supplementary S5 description Study Population and Data Collection**

This national cohort study relies on data from the Korean Health Insurance Review and Assessment Service - National Sample Cohort (HIRA-NSC). The Korean National Health Insurance Service (NHIS) selects samples directly from the entire population database to prevent non-sampling errors. Approximately 2% of the samples (one million) were selected from the entire Korean population (50 million). This selected data can be classified at 1,476 levels (age [18 categories], sex [2 categories], and income level [41 categories]) using randomized stratified systematic sampling methods via proportional allocation to represent the entire population. After data selection, the appropriateness of the sample was verified by a statistician who compared the data from the entire Korean population to the sample data. The details of the methods used to perform these procedures are provided by the National Health Insurance Sharing Service [[14](#_ENREF_14)]. This cohort database included (i) personal information, (ii) health insurance claim codes (procedures and prescriptions), (iii) diagnostic codes using the International Classification of Disease-10 (ICD-10), (iv) death records from the Korean National Statistical Office (using the Korean Standard Classification of disease), (v) socio-economic data (residence and income), and (vi) medical examination data for each participant over a period ranging from 2002 to 2013.

Because all Korean citizens are recognized by a 13-digit resident registration number from birth to death, exact population statistics can be determined using this database. It is mandatory for all Koreans to enroll in the NHIS. All Korean hospitals and clinics use the 13-digit resident registration number to register individual patients in the medical insurance system. Therefore, the risk of overlapping medical records is minimal, even if a patient moves from one place to another. Moreover, all medical treatments in Korea can be tracked without exception using the HIRA system. In Korea, notice of death to an administrative entity is legally required before a funeral can be held. Causes of death and date are recorded by medical doctors on a death certificate.

**Meteorological Data**

Temperature (˚C), Relative humidity (%), were Spot atmospheric pressure (hPa) data were obtained from the meteorological administration. It was measured by automated synoptic observing system (ASOS) and manually in 94 places hourly. Quality was controlled following quality inspection manual (<https://data.kma.go.kr/cmmn/main.do>).

SO_2_ (ppm), NO_2_ (ppm), O_3_ (ppm), CO (ppm), and PM_10_ (μg/m3) were data were obtained by the ministry of environment. It was measured by ASOS in 273 place over the country hourly. Quality was controlled following air pollution quality control manual (<http://www.me.go.kr/home/web/index.do?menuId=10259>). We used daily mean values.
